# Facile and Quantitative Method for Estimating the Isolation Degree of Cellulose Nanocrystals (CNCs) Suspensions

**DOI:** 10.3390/ma14216463

**Published:** 2021-10-28

**Authors:** Minwoo Lee, Minhaeng Heo, Hyunho Lee, Jihoon Shin

**Affiliations:** 1 Center for Environment & Sustainable Resources, Korea Research Institute of Chemical Technology (KRICT), 141 Gajeong-ro, Yuseong-gu, Daejeon 34114, Korea; mhheo@krict.re.kr (M.H.); hyunho@krict.re.kr (H.L.); 2 Department of Advanced Materials & Chemical Engineering, University of Science & Technology (UST), 217 Gajeong-ro, Yuseong-gu, Daejeon 34113, Korea

**Keywords:** cellulose nanocrystals, high-pressure homogenization, turbidity, surface charge, fractionation

## Abstract

The isolation degree of cellulose nanocrystals (CNCs) suspensions calculated from the amount of sediments obtained with the centrifugation method can be estimated with turbidimetry, surface charge and dispersion analysis of the CNCs suspension. Three different types of raw cellulosic materials were used and carried out with an acid hydrolysis and mechanical disintegration. As the number of high-pressure homogenizer treatments increased, the isolation degree of CNCs from microcrystalline cellulose (MCC) increased from 2.3 to 99.6%, while the absorbencies from turbidimetric measurement of the CNCs suspension decreased, from 2.6 to 0.1 Abs units. Furthermore, the surface charges based on zeta potential measurements of the CNCs suspensions increased from −34.6 to −98.7 mV, but the heights of sediments from the CNCs suspensions were reduced, from 4.01 to 0.07 mm. Similar results were obtained for CNCs from softwood pulp (SWP) and cotton pulp (CP). These results show a direct correlation between yield, turbidity, surface charge and sedimentation of CNCs suspensions. Their correlation indices (0.9) were close to a maximal value of 1. This approach can be suggested as a facile and rapid estimation method for CNCs manufacturing process.

## 1. Introduction

Cellulose nanocrystals (CNCs) are among the most attractive types of renewable nanoscale materials, with a highly ordered and tightly packed crystalline structure due to strong hydrogen bonds. They are mainly extracted from wood or cotton pulp, industrial papermaking waste and non-edible biomass residue, which are appropriate resources given the economic stakes and available supply. CNCs have attracted exponentially growing interest for the last 20 years due to their numerous applications, which include reinforcing agents [1,2,3,4,5,6], biomedical devices [7,8,9], rheological modifiers [10,11], flexible electronics or sensors [12,13,14] and replacements of conventional nanomaterials (e.g., carbon nanotubes, graphene, nanoclay, metal and inorganic nanoparticles) [15,16]. Because of these wide applications, there is a substantial demand for research on the commercial development of CNCs, including manufacturing processes and facilities. However, until early 2012, CNCs were still mainly prepared several grams at a time using laboratory apparatus in a university. In recent years, Canada has become the world leader in pilot units and commercial plants for the large-scale production of CNCs, with four different companies (CelluForce, Alberta Innovates, BioVision and US Forest Product Lab). The other pilot units are located in the USA [17].

The efficient production of CNCs is influenced by several factors, such as the type of raw material, chemical consumption or recycling, hydrolysis conditions, equipment development and new methods [18,19]. Given the potential variability of the factors mentioned above, industrial processing is not that simple, and still requires research as well as synergy between engineers and researchers. In this regard, methods that can quickly monitor and step-count the degree of hydrolysis (or degree of isolation from micron to nano-size) in real-time are needed for engineers and other workers. For example, if the chemical treatment (mainly acid hydrolysis) used to isolate the cellulosic materials proceeds without a problem, just one or a few cycles of mechanical disintegration process will be required. If not, a higher dosage of mechanical treatment energy will be required, or it might not even reach nano-levels.

When a mechanical disintegration process is conducted on hydrolyzed cellulose, an electrostatically stable colloidal suspension is produced, by exposing sulfate half ester (OSO_3_^-^) groups on the CNCs surfaces, which are able to sufficiently penetrate into the cellulose molecules during hydrolysis. The suspension becomes more transparent with increasing cycles of mechanical disintegration. This change in transparency indicates that almost all of the cellulose fractions have reached nano-scale, and thus have lower light scattering potential [20,21].

Various spectrophotometric techniques have been employed to estimate the degree of cellulose nanofiber material fibrillation, as well as predict the size of their products. Shimizu et al. examined the partial relationship between the widths of nanocellulose and turbidities in a water dispersion and found that the results were well correlated with the AFM-measured thickness [22]. Yano et al. showed that the light transmittance of composites prepared with fibrillated pulp and acrylic resin increased with increasing degree of fibrillation [23].

Generally, reinforcing materials suffer a loss of transparency due to the increase in light scattering of their composite, but the refractive index of cellulose nanofibers is too small to affect the transparency of their composites. Aulin et al. determined the light transmittance of two carboxymethylated microfibrillated (MFC) films prepared by dispersion casting from aqueous dispersions homogenized once and 10 times, respectively [24]. The MFC film prepared from a dispersion homogenized 10 times was transparent (90%) compared with the film prepared from a dispersion homogenized once, which appeared opaque (25%). This means that the degree of nanofibrillation/dispersion has a decisive effect on the microstructure and optical properties of the films.

Chinga-Carrasco et al. prepared a series of MFC suspensions by mixing two MFC qualities; one was a highly fibrillated material (TEMPO-oxidized MFC), the other had an insufficient degree of fibrillation (homogenized MFC without any chemical pretreatment) [21]. Films with partially unfibrillated fractions were more opaque than highly fibrillated fractions. They also mentioned that an optical method can be used in online production lines to quantify and assess the degree of fibrillation in a given material, for process control. However, those evaluation methods were only used to estimate the width of the nanocellulose, or to assess the characteristics of the final products with added nanocellulose, such as composites, papers and films. For those reasons, they are unsuitable for conducting an accompanying simple and useful measurement, to determine the amount of nano-sized fraction isolated from the macro cellulose fraction during mechanical treatment in an online process.

The typical procedure used to fractionate cellulose particles in the nano size range from mechanically isolated treated cellulosic materials is the centrifuge separation method, which can be effectively used to remove any larger agglomerates or some un-isolated coarse fragments from the final cellulose nanoparticles suspension. The centrifugal separation method is based on the rate of settling of particles in a liquid at rest under a gravitational or centrifugal field. Larger particles have a greater tendency than smaller particles of the same material to settle to the bottom of the dispersion. Cellulose nanoparticles prepared by acid hydrolysis and mechanical isolation, because of their small size and high negative surface charge, are electronically stable in an aqueous suspension, which prevents their sedimentation during centrifugation. At the same time, since the small dimensions (width less than 100 nm) of the nanoparticles lower their light scattering potential, this in turn reduces the opacity of the aqueous suspension and thus increases its light transmittance [25]. This fundamental optical phenomenon could be a way to demonstrate and confirm the potential of relatively simple methods, for assessing the degree of isolation of cellulosic materials. This method, using optical devices such as a spectrophotometer and/or turbidimeter, is simple and can quantify the nano-sized cellulose fractions in suspension. Such a system would have significant potential in real-time manufacturing process control.

Generally, it has been demonstrated that increasing surface charge values will boost acceleration effects during the fibrillation of cellulosic materials, i.e., the zeta potential values reveal a higher negative charge depending on the degree of fibrillation or isolation of the CNF or CNCs [26,27]. Zhao et al. reported that the refining process results in physical changes of the fiber cell wall structures, including fibrillation and fiber shortening [28]. This can increase the fiber’s surface charge by increasing the specific surface area and/or by changing the chemical compositional distribution on the fiber surfaces to some extent. Banavath et al. determined that the fiber surface charge increased linearly with an increase in refining level. As the refining process opens up the fiber surface, the fiber surface charge increases [29]. It should be noted that increasing the specific surface area of the pulp increased the fiber surface charge. When refining produced a large fiber surface area it resulted in a proportional increase in fiber surface charge.

In this work, we first prepared CNC samples extracted using the conventional sulfuric acid hydrolysis method from different types of cellulosic sources (MCC, wood and cotton pulp). Hydrolyzed cellulosic materials were isolated using a high-pressure homogenizer (HPH) for a number of passes, and an evaluation of optical and surface charge properties of CNC suspensions was carried out. We showed that the turbidity and zeta potential values of the aqueous suspensions, including for different CNC quantities, can be utilized in a simple and fast method to estimate the degree of isolation (the yield of CNCs in aqueous suspension) when using the centrifugal separation method.

## 2. Results and Discussion

### 2.1. Mass Fraction Separation of CNCs Aqueous Suspensions

**Scheme 1:**
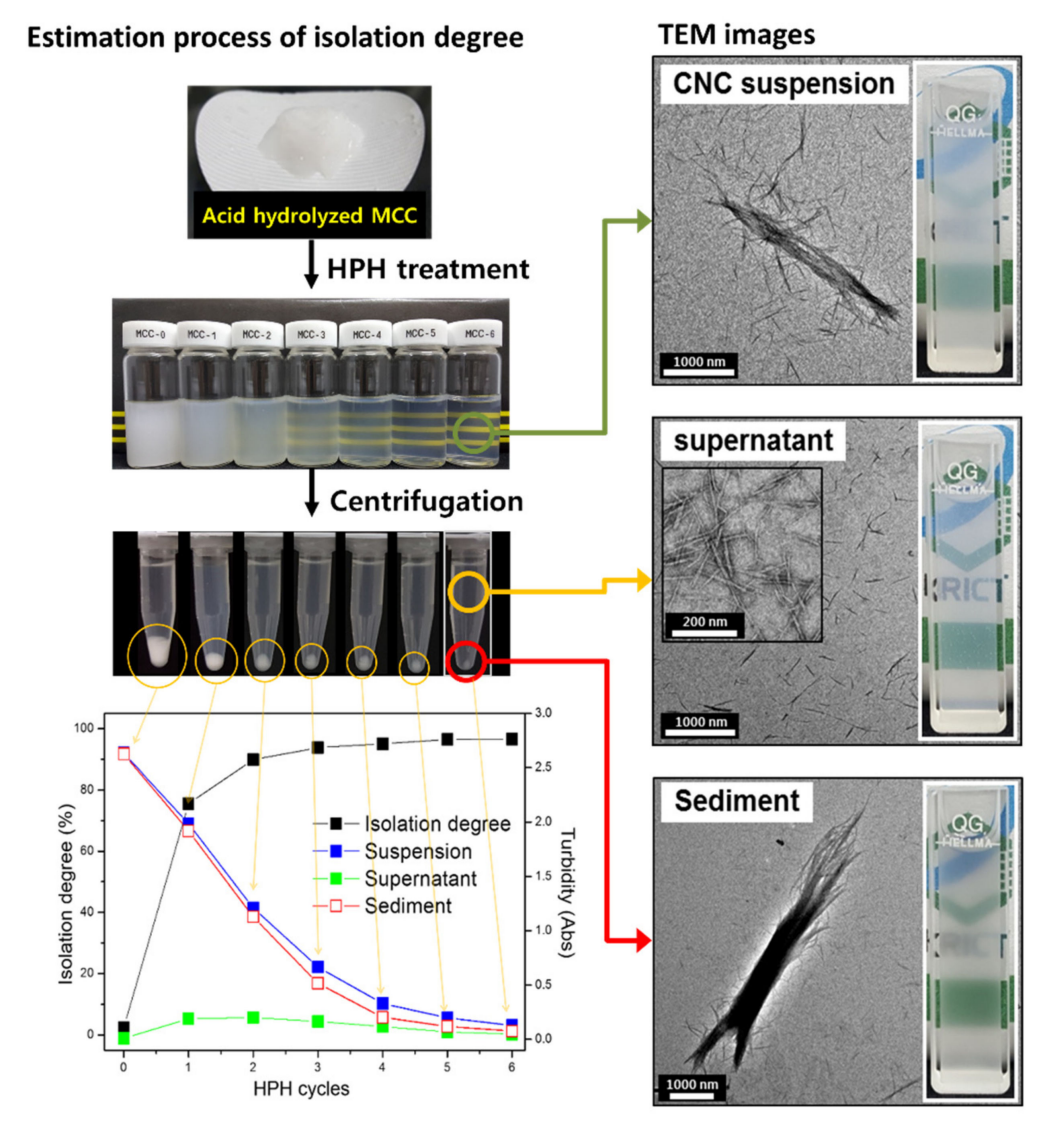
Schematic diagram for estimation of isolation degree of CNCs suspensions from MCC according to high-pressure homogenizer (HPH) treatment with turbidimetry.

We developed a facile method for estimating the isolation degree of CNCs from the turbidity of their suspensions. We determined that the turbidity of the CNCs suspensions decreased significantly as the mechanical disintergration treatment was increased. Several studies have described how acid hydrolysis generates negative charges that induce repulsive forces between cellulose microfibrils, which helps loosen the microfibrils’ cohesion, due to hydrogen bonding, and degrades the disordered regions of the cellulose microfibrils, making them more scissile [30]. The mechanical disintergration leads to a decrease in the amount of settling fractions separated from the CNCs aquous suspensions by centrifugal sedimentation treatment. This increased the degree of CNCs isolation in suspension and decreased turbidity (Scheme 1). The tiny fractions stabley dispersed in the suspension ranged in size from several tens to hundreds of nanometers.

**Figure 1:**
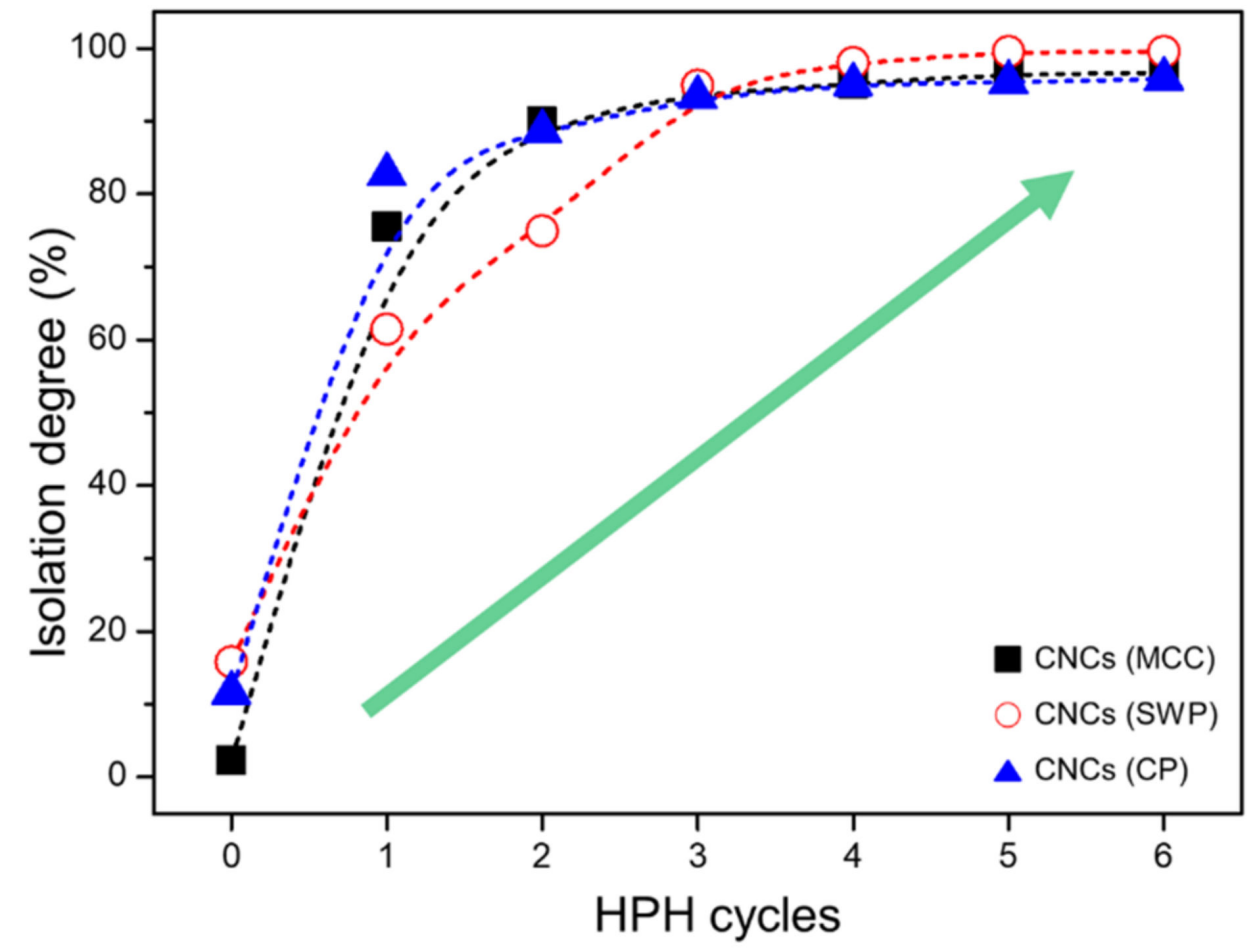
The isolation degree of CNCs suspensions from various cellulose materials following mechanical treatment. Square: CNCs (MCC); circle: CNCs (SWP); triangle: CNCs (CP).

Many studies have been conducted on the separation of nano-sized fractions from aqueous suspensions containing nano- and micro-sized cellulose particles using filtration or centrifuge methods [31,32,33]. However, it is difficult to separate nano-sized cellulose crystals by filtration methods because the nanocrystals can pass through the membrane due to the CNC structure’s aspect ratio, which can be rod-like. During centrifugation, the large fractions that did not reach nano-size will precipitate to the bottom of the tube first because they have a higher velocity than the nano-sized fractions. In the supernatant after centrifugation at more than 195 relative centrifugal force (RCF), the nano-sized fractions have widths of less than 100 nm [34]. The isolation degree of CNCs suspensions was calculated from the amount of sediments in the suspensions. Importantly, the isolation degree of the CNCs suspension is associated with yield of the resultant CNCs. The isolation degree of the CNCs suspension increased exponentially with the cycles of high-pressure homogenizer (HPH) treatment. Specifically, the MCC, SWP and CP increased from 2.3, 15.8 and 11.6 to 96.6, 99.6 and 95.8, respectively, after six cycles of HPH treatment (Figure 1).

### 2.2. Optical Properties of CNCs Aqueous Suspensions

**Figure 2:**
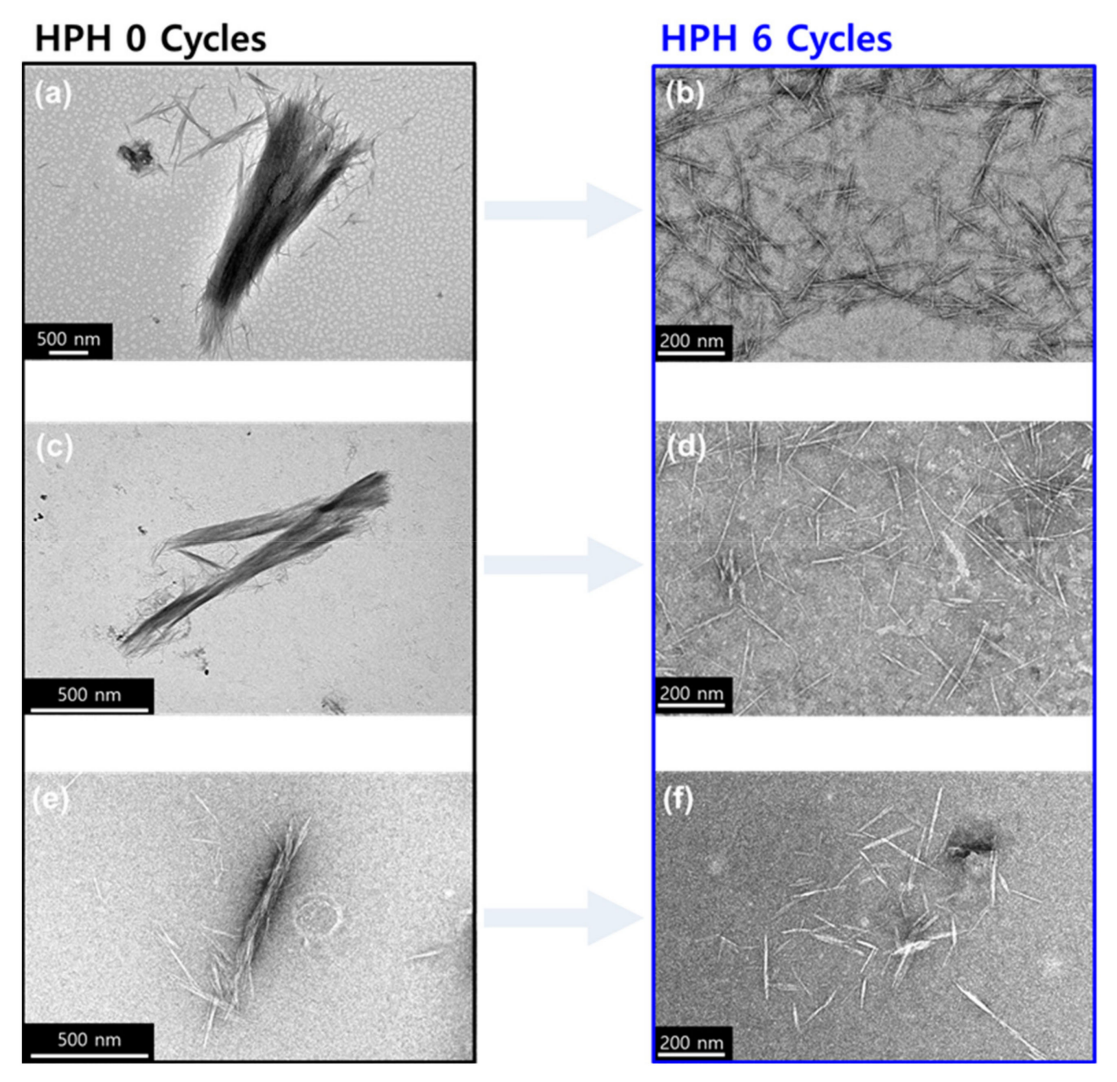
TEM images of negatively stained CNCs obtained by sulfuric acid hydrolysis, from three different materials, with or without mechanical treatment: (**a**) MCC-untreated, (**b**) MCC-6 cycles, (**c**) SWP-untreated, (**d**) SWP-6 cycles, (**e**) CP-untreated, (**f**) CP-6 cycles.

We investigated the influences of mechanical treatments that resulted in down-sizing the morphologies of the cellulose fractions in suspensions. Representative TEM and AFM images (Figure 2 and Figure S1), showing before and after pictures of the cellulose fractions, indicate that mechanical disintegration was accomplished by the HPH treatment ((Figure 2a,c,e): cellulose hydrolyzed by sulfuric acid using an acid-to-cellulose ratio of 16.38 wt % for 2 h and (Figure 2b,d,f): nano-sized fractions showing the mechanical disintegration of the hydrolyzed cellulose after 6 cycles of HPH treatment). After 6 cycles of HPH treatment, the nano-sized fractions appeared as rod-like cellulose crystals about 10–20 nm in width and 300–400 nm in length. On the other hand, the fractions of untreated mechanical disintegration remained as micro-sized cellulose bundles, as shown in Figure 2a,c,e. Multiple studies and commercial applications have demonstrated that high-pressure homogenizers are very effective for the disintegration and delamination of acid-hydrolyzed cellulose [34,35].

**Figure 3:**
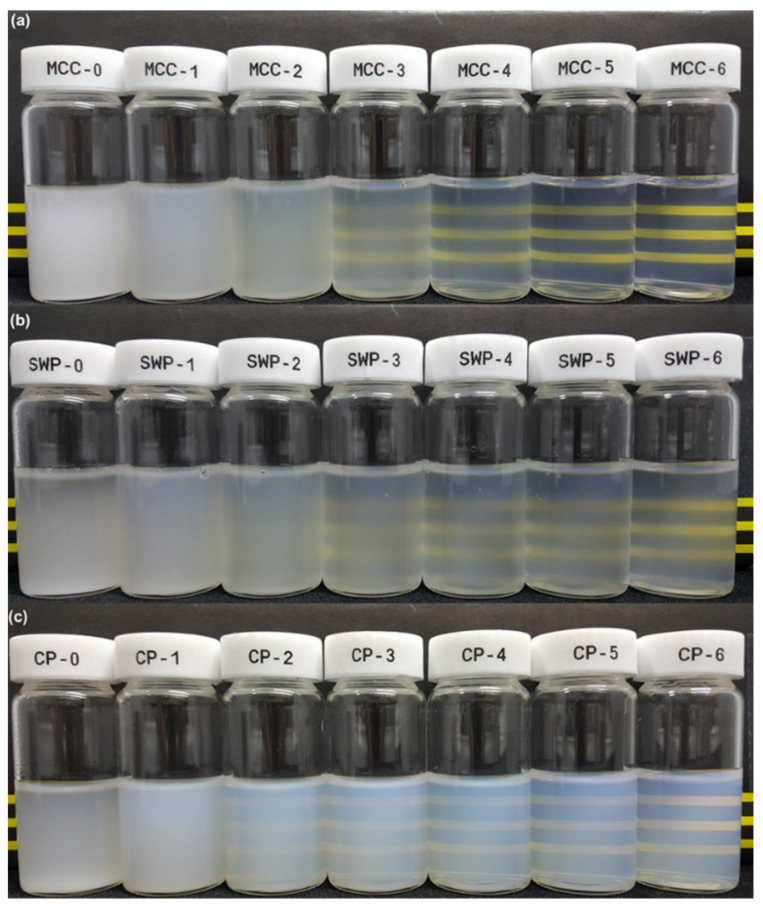
Photograph of CNCs suspensions with changing turbidity due to increased mechanical treatment: (**a**) CNCs (MCC), (**b**) CNCs (SWP), (**c**) CNCs (CP).

Another very interesting relationship between the optical refractive properties of CNCs suspensions and particle size was investigated by turbidity and light transmittance analyses. The results in Figure 3 clearly demonstrate the dependence of light transmittance on particle size in the CNCs suspension. When the size of the cellulose fractions was smaller than half of the visible light wavelength (about 350 nm), the light transmittance of the suspensions increased. It seems that the nanocrystals in the suspensions were too small to reflect light, and were homogeneously dispersed in the suspension without any agglomeration [19,36].

In Rayleigh light scattering theory, the intensity of the scattered light depends on particle size, but when the particle sizes are larger than the wavelength (visible light wavelength, 600 nm in our study), Mie scattering is dominant [37,38]. Mie scattering occurs when the diameters of the particulates are similar to or larger than the wavelengths of the scattered light, and also increase exponentially with increasing particle size. It is also noted that the Mie scattering of nanoparticles becomes much more serious with a change in fraction size, and this suggests that the yield of cellulose nanocrystals can be predicted by measuring light transmittance. The reason that the Mie scattering theory can be applied to this study is that if the particles in the CNCs suspension are larger than the visible light wavelength, scattering will occur a lot and the turbidity will increase. It is also noted that the correlation with the amount of unfibrillated cellulose fractions and turbidity of the CNCs suspension can be observed.

**Figure 4:**
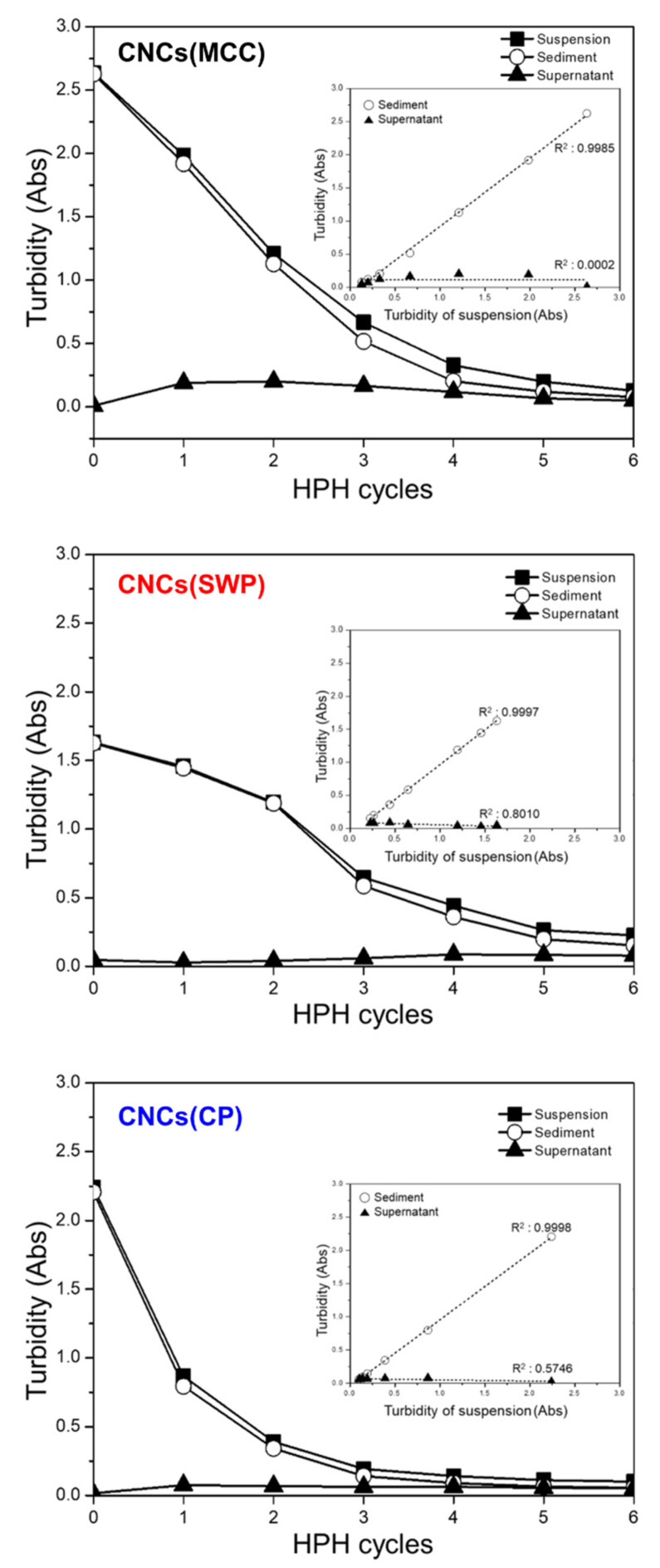
Turbidity curve of the suspension, sediment and supernatant of CNCs dispersions according to number of HPH treatments. (inset) Comparison of the turbidity of sediment and supernatant and the turbidity of the suspension of CNCs.

Based on the aforementioned theory and available research, we evaluated the turbidities of the CNCs suspensions following centrifugal treatment, which were divided into supernatants and precipitates, using a UV-Vis spectrometer (Figure 4). In order to know whether the turbidity of the CNCs suspension affects the supernatant or the precipitate, pure water was added to the precipitate with high solids instead of the supernatant after centrifugation to adjust the volume to the same as the original CNCs suspension. The diluted precipitates containing large size fractions caused a lot of scattering, and showed a level similar to the turbidities of the CNCs suspensions, while the supernatants were very transparent. In other words, because the turbidities of precipitates have a high linear correlation with that of the CNCs suspensions (inset), by measuring the turbidities of the CNCs suspensions, the turbidity curve can be calculated by inversely calculating the yield of nanocrystals.

### 2.3. Surface Charge and Dispersion Stability of CNCs

**Figure 5:**
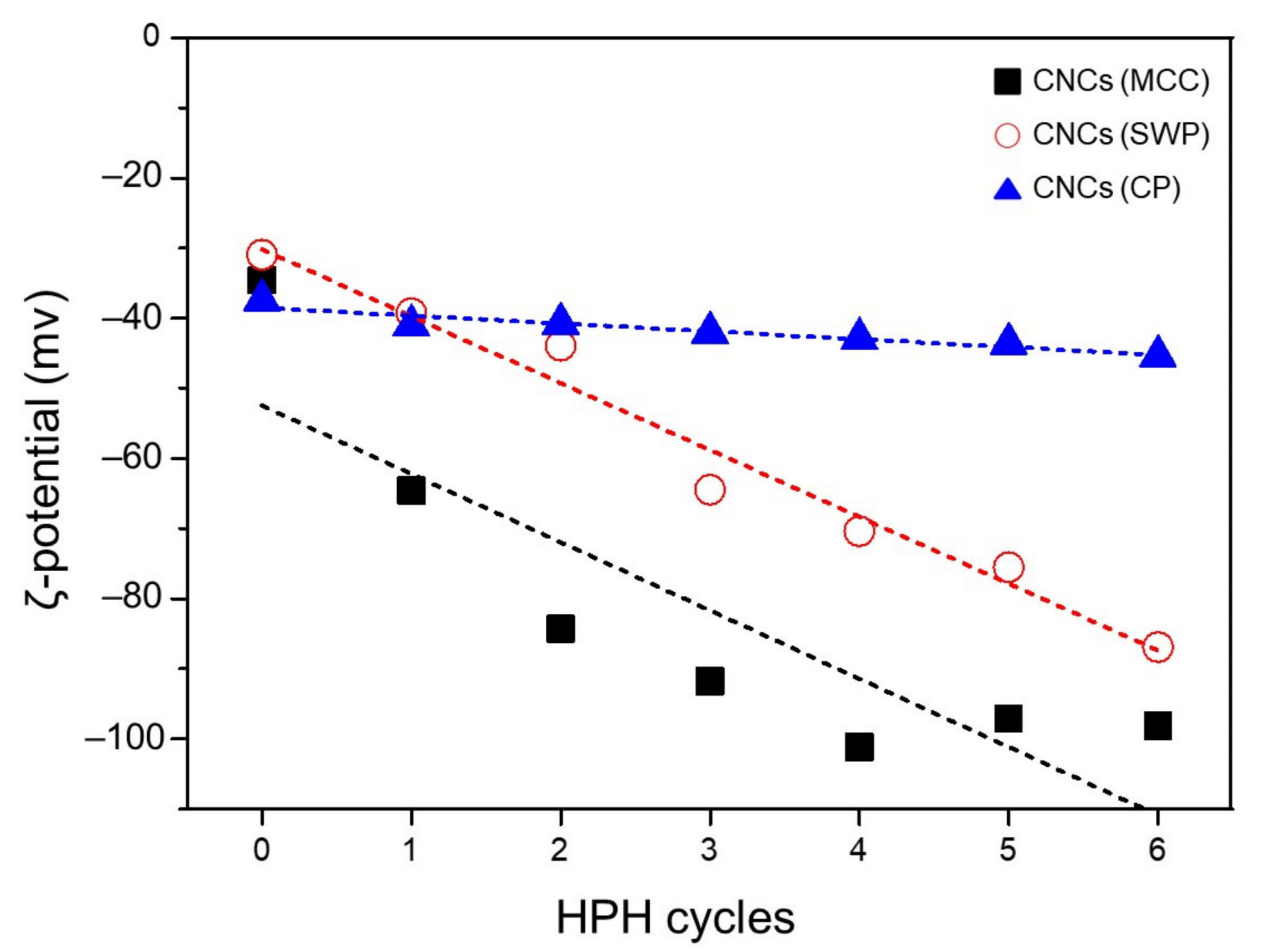
Zeta potential of CNCs from various materials (squares: CNCs (MCC) of 1.0 wt %; circles: CNCs (SWP) of 0.5 wt %; triangles: CNCs(CP) of 1.0 wt %.).

The stability of the CNCs suspensions, which is influenced by the electrostatic repulsion of the negative charged surfaces, was estimated by measuring zeta potential (Figure 5). Surface charge is one of the most important factors affecting the quality of CNCs suspensions, and is usually measured using a Zetasizer. The zeta potential values of the CNCs suspensions from MCC (−34.6 ± 0.3 to −98.7 ± 1.0 mV), Softwood pulp (−31.0 ± 1.4 to −84.0 ± 1.5 mV) and Cotton pulp (−37.4 ± 0.4 to −45.4 ± 0.4 mV) (Table 1) satisfied the minimum threshold (±30 mV) for a high degree of stability considering their absolute values. The absolute zeta potential values of the CNCs suspensions gradually increased with the number of mechanical treatments, as shown in Figure 5. Several studies have also shown similar trends, and reported that the absolute zeta potential values increased with increasing mechanical treatment due to the fibrillation of the fiber surface [28,39].

An increase in the surface area or the presence of exposed sulfate groups on the cellulose surface due to the mechanical treatments can increase the absolute zeta potential value. Interestingly, the surface charge of the CNCs (CP) hardly changed even with the increase in mechanical treatment, compared to the other two samples. This indicates that the level of ordering of the crystalline region in the cotton pulp is higher relative to wood pulp or regenerated cellulose, suggesting that the acid hydrolysis of a more ordered cellulose does not ensure the sufficient introduction of sulfate groups (Table 2). Despite the low surface charge, the isolation degree was ironically high, due to the high degree of crystallinity. Udomkichdecha et al. reported that crystallinity has a positive correlation with the fibrillation of fibers because fibrillation is affected by oriented crystalline regions [40]. Since the cotton pulp used had a high crystallinity of 91.6%, even though there were less sulfate groups introduced, fibrillation by mechanical disintegration occurred easily.

**Figure 6:**
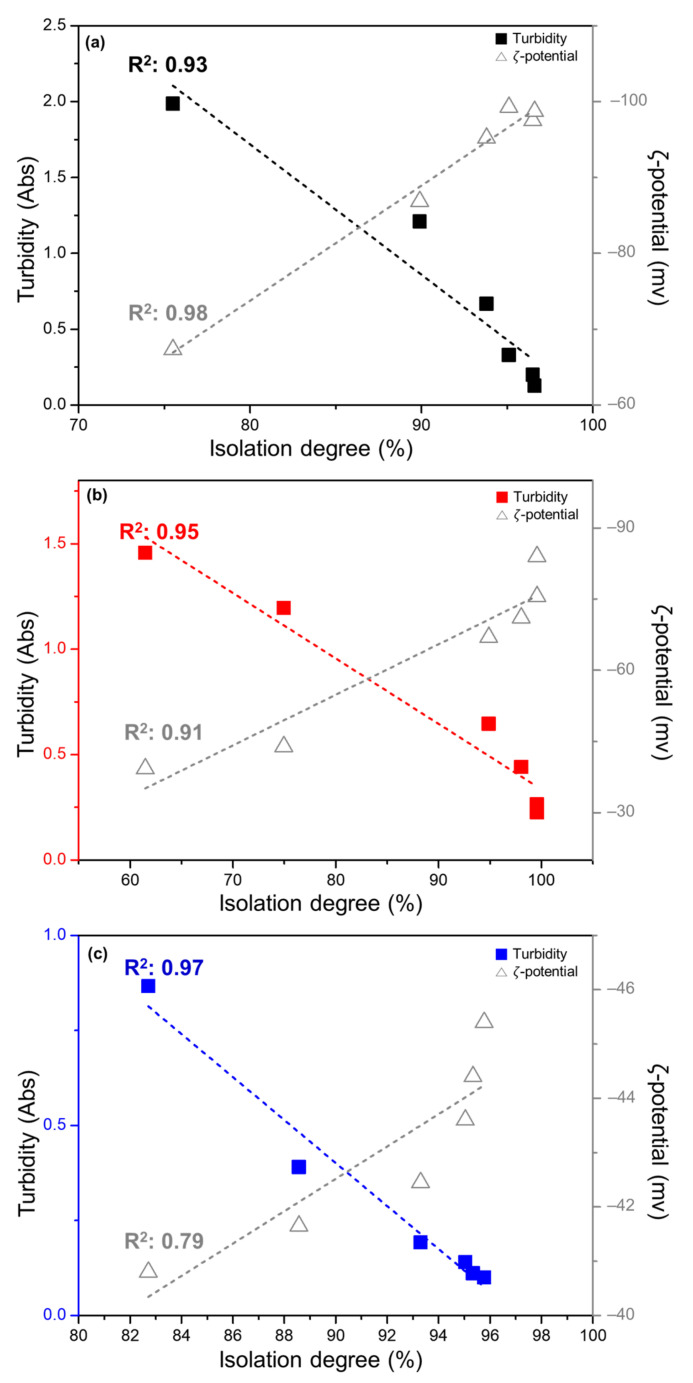
Comparison of turbidity, zeta potential and isolation degree of the various CNCs suspensions. (**a**): CNCs (MCC), (**b**): CNCs (SWP), (**c**): CNCs (CP).

It was confirmed that the isolation degree, turbidity and zeta potential of each of the CNCs suspensions tended to change depending on mechanical disintegration. The values from the three different evaluation methods showed a fairly intimate relationship with each other, and more specifically, the isolation degree of the CNCs suspensions had a strong linear correlation with turbidity and zeta potential, as shown in Figure 6. The R-squared values for isolation degree and turbidity were 0.93, 0.95 and 0.97 for the MCC, SWP and CP respectively. The isolation degree of the CNCs suspensions can be calculated using the amount of sediments (macro cellulose fractions) in the suspensions separated from the supernatant by centrifugation, as well as the turbidity, which also affected by the large fractions in the suspensions. The two evaluation methods can be seen to be strongly correlated. Furthermore, there is a high correlation between the isolation degree and zeta potential of the CNCs suspensions. The correlation coefficients (R^2^) were 0.98, 0.91 and 0.79 for the MCC, SWP and CP, respectively. This is because, as the number of mechanical disintegrations inducing the fibrillation of cellulose increases, the number of nano-sized fractions increases and the number of exposed sulfate half ester groups increases.

A dispersion stability analyzer (LUMISizer, GmbH) was used to evaluate sedimentation of colloidal dispersions within a short time, and revealed there was a direct correlation between the isolation degree of the CNCs suspensions and the height of the sediment, as shown Figure S2. R squared values were between 0.96 and 0.99 for the linear correlation between isolation degree and the height of sediments. This result indicates that the isolation degree of the CNCs suspension can be also evaluated effectively with LUMISizer (Figure S3).

### 2.4. Size Distribution of the CNCs

**Figure 7:**
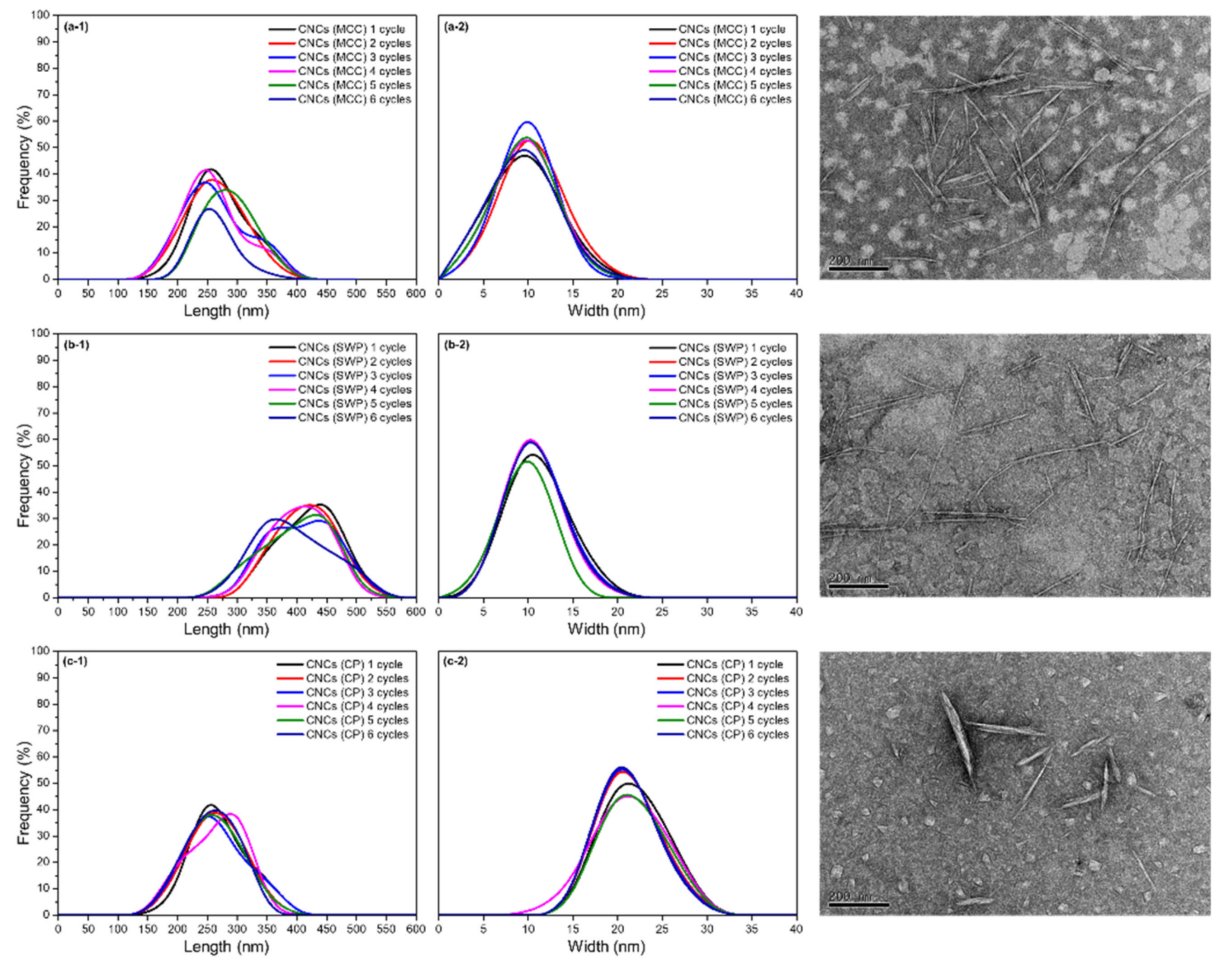
Size distribution histograms and TEM images of supernatant: (**a-1**, **b-1**, **c-1**) length of CNCs (MCC, SWP, CP), (**a-2**, **b-2**, **c-2**) width of CNC (MCC, SWP, CP).

The dimensions of the cellulose nanocrystals in the suspensions were determined by TEM images of more than 50 individual particles, as shown Figure 7. The average values are presented in Table 1. All of the CNCs samples exhibited a relatively narrow distribution of lengths, between 200–350 nm (both MCC and CP) and 300–500 nm (SWP). The widths of the CNCs also had a narrow distribution with particles that ranged from 5–15 nm (both MCC and SWP) and 15–25 nm (CP).

For the CNCs (CP), the width was defined as the largest measured dimension because cotton has the highest crystallinity and is strongly packed compared to wood or regenerated cellulose. Still, the nano-sized cellulose crystals present in the supernatant of the suspensions all had a uniform size, and many studies have reported that CNCs of a uniform size were obtained by the centrifugation method [41,42]. Bai et al. reported that the sediments in CNCs suspensions with a size of more than 280 nm were almost precipitated after centrifugation at more than 1751 RCF, and that the small size CNCs were uniformly dispersed in the supernatant [43]. Although it is possible to accurately observe the morphology of CNCs using TEM measurement, it does not accurately represent all of the CNCs. The measurement is also difficult, and takes a long time, so it is difficult to use TEM as a CNCs product manufacturing control index [22]. By identifying the correlation between turbidity, yield and zeta potential of CNCs suspensions, the present study will make it possible to effectively manage real-time manufacturing processes.

The sizes of nanoparticles have been widely measured using dynamic light scattering (DLS). This is a comparatively cheap and useful method for measuring size distributions, but DLS does not measure the dimension of particles directly, like TEM or any other type of microscopy. It measures the hydrodynamic diameter of nanoparticles [44]. Actually, DLS is inadequate when measuring the dimensions of nanocellulose, because of the high aspect ratio of nanocelluloses, but it is possible to measure the uniformity of the overall sample size, unlike TEM measurement, which only measures local parts of the sample.

**Figure 8:**
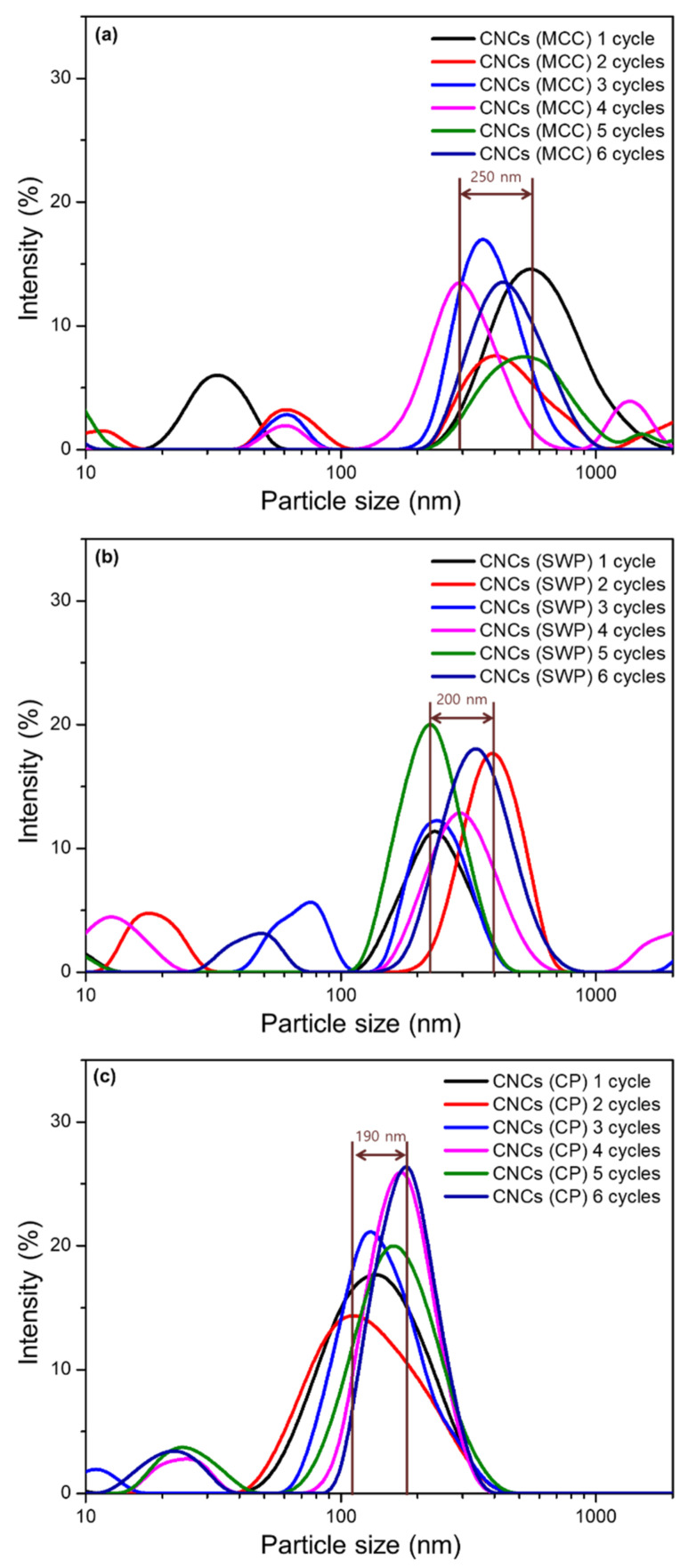
The DLS intensity distribution for diverse CNCs suspensions according to HPH treatment: (**a**) Isolated CNCs from MCC, (**b**) isolated CNCs from SWP, (**c**) isolated CNCs from CP.

The CNCs in supernatant have entirely narrow distribution and the values of between maximum and minimum size were 250, 200 and 190 nm of MCC, SWP and CP, respectively (Figure 8). Furthermore, uniform CNCs were obtained with only one pass of the high pressure homogenzier treatment. In particular, as mentioned above, CNCs (CP) had the highest crystallinity, which can lead to more fibrillation compared other cellulose sources, ensuring a more uniform size.

The average length of the CNCs (MCC), CNCs (SWP) and CNCs (CP) ranged from 275.9 ± 40.7 to 233.4 ± 36.8 nm, 486.8 ± 93.7 to 385.2 ± 123.3 nm and 291.0 ± 75.5 to 274.3 ± 73.9, respectively (Table 1). Studies in the literature using TEM analysis have reported the particle size of CNCs from microcrystalline cellulose and wood as ranging from 50 to 500 nm in length and 5 to 20 nm in width, and cotton as ranging from 50 to 300 nm in length and 10 to 50 nm in width [41,46,47,48].

After at least three HPH treatments, the width, length and isolation degree of the CNCs exhibited uniform and constant values regardless of the number of further treatments. The width and length of the CNCs were uniform regardless of the number of HPH treatments, and more than 90% isolation degree could be obtained in all CNCs samples when there were at least three HPH treatments. The turbidity of the CNCs (MCC), CNCs (SWP) and CNCs (CP) suspensions ranged from 2.635 ± 0.000 to 0.128 ± 0.001, 1.633 ± 0.004 to 0.226 ± 0.001, and 2.241 ± 0.001 to 0.100 ± 0.000, respectively. As the number of HPH treatments increased, the turbidity of the CNCs suspensions gradually decreased. The absolute value of zeta potential gradually increased as the sulfate half ester group exposure increased with HPH treatment. The zeta potential value of the CNCs (MCC), CNCs (SWP) and CNCs (CP) suspensions ranged from −34.6 ± 0.3 to −98.7 ± 1.0, −31.0 ± 1.4 to −84.0 ± 1.5 and −37.4 ± 0.4 to −45.4 ± 0.4, respectively.

For the three samples, it can be seen that the isolation degree, turbidity, surface charge and stability of the CNCs suspension showed a trend as the HPH treatment increased. However, in the case of CNCs (SWP), the sample of HPH treatment applied six times showed a slightly lower stability than the sample of HPH treatment applied five times because the length of CNCs was larger. As mentioned above, since the high-pressure homogenizer treatment showed a fractionation degree of 90% or more with only three times, some deviation of data may be seen in the treatment three times or more.

Cellulose is a linear polymer composed of D-glucose units linked by β-1,4-glycosidic bonds. The hydroxyl groups in cellulose are involved in a lot of intra- and intermolecular hydrogen bonds, resulting in various ordered crystalline arrangements [51]. The crystallinity index (CI) values of CNCs (MCC), CNCs (SWP) and CNCs (CP), were 82.0, 85.2 and 91.6%, respectively (Table 2). CNCs (CP) had lower sulfate half ester content than the other CNCs materials even under the same hydrolysis conditions because the strongly packed crystalline region of the cotton materials distrubs acid hydrolysis [52]. There were variations in the degree of polymerization (DP) depending on the original materials. The highest DP (1266) was found for SWP, followed by those for CP and MCC (DPs of 1071 and 348), respectively.

## 3. Conclusions

This study developed a facile and quantitative method for estimating the isolation degree of cellulose nanocrystals (CNCs) suspensions using turbidimetry, surface charge and dispersion analysis. Three different types of CNCs suspensions were produced from microcrystalline cellulose (MCC), softwood pulp (SWP) and cotton pulp (CP) using sulfuric acid hydrolysis and high-pressure homogenization. By identifying the correlation between the turbidity, yield and zeta potential of CNCs suspensions, this study will make it possible to effectively manage real-time manufacturing processes. The results demonstrated the possible commercial applications of the correlation between turbidity, yield and zeta potential of CNCs suspensions. Cellulose nanocrystals (CNCs) with uniform sizes of 10 to 20 nm in width and 200 to 500 nm in length were efficiently obtained using high-pressure homogenizer treatment, and the isolation degree of CNCs could be predicted by quantifying large-sized fractions separated by centrifugation. There was a slight difference depending on the crystallinity of the raw material itself, but it was found that the optical transparency, surface charge and yield of the CNCs suspensions produced by mechanical disintegration are highly correlated with each other, with a correlation coefficient of 0.9 or more. Cellulose nanocrystals have great potential for use as a renewable material, and can be extracted from abundant lignocellulosic biomass resources. Therefore, it is expected that this innovative study will lay the foundation for further application development, by developing a system that improves the management of the cellulose nanocrystals production process and ensures uniform quality.

## Figures and Tables

**Table 1:** Characteristics of CNCs suspensions from diverse cellulosic materials.

CNCs Samples ^a^	Width (nm) ^b^	Length (nm) ^b^	Isolation Degree of CNCs(%) ^c^	Turbidity (Abs) ^d^	ζ-Potential (mv) ^e^	Stability (%) ^f^
MCC0	58.2 ± 25.5 ^g^	18.4 ± 7.7 ^g^	2.3 ± 1.4	2.635 ± 0.000	−34.6 ± 0.3	0.0
MCC1	9.5 ± 2.6	275.9 ± 40.7	75.5 ± 0.6	1.986 ± 0.003	−67.3 ± 1.0	78.6
MCC2	10.8 ± 2.1	266.5 ± 41.3	89.9 ± 0.1	1.210 ± 0.002	−86.9 ± 1.3	92.6
MCC3	9.9 ± 1.8	265.3 ± 45.7	93.8 ± 1.2	0.666 ± 0.002	−95.2 ± 2.1	98.0
MCC4	10.1 ± 2.2	259.5 ± 43.7	95.1 ± 1.0	0.329 ± 0.001	−99.3 ± 3.9	97.9
MCC5	9.3 ± 1.9	269.9 ± 40.1	96.5 ± 0.8	0.200 ± 0.001	−97.5 ± 0.0	97.5
MCC6	9.3 ± 2.4	233.4 ± 36.8	96.6 ± 1.0	0.128 ± 0.001	−98.7 ± 1.0	98.3
SWP0	95.6 ± 28.5 ^g^	28.7 ± 10.0 ^g^	15.8 ± 1.0	1.633 ± 0.004	−31.0 ± 1.4	0.0
SWP1	10.6 ± 3.0	486.8 ± 93.7	61.5 ± 0.8	1.457 ± 0.001	−39.3 ± 0.6	37.7
SWP2	12.7 ± 2.2	438.6 ± 95.5	75.0 ± 0.2	1.196 ± 0.001	−43.9 ± 0.8	73.6
SWP3	10.4 ± 3.8	423.8 ± 128.3	94.9 ± 0.5	0.646 ± 0.000	−67.0 ± 1.1	88.6
SWP4	10.7 ± 4.4	399.5 ± 138.6	98.0 ± 0.3	0.442 ± 0.000	−71.1 ± 0.1	93.0
SWP5	10.6 ± 3.6	384.5 ± 103.2	99.6 ± 0.7	0.264 ± 0.000	−75.5 ± 2.1	95.1
SWP6	10.1 ± 3.7	385.2 ± 123.3	99.6 ± 0.1	0.226 ± 0.001	−84.0 ± 1.5	92.5
CP0	54.9 ± 24.6 ^g^	17.5 ± 6.0 ^g^	11.6 ± 0.3	2.241 ± 0.001	−37.4 ± 0.4	0.0
CP1	21.5 ± 3.2	275.2 ± 76.6	82.7 ± 0.4	0.867 ± 0.001	−40.8 ± 0.1	95.2
CP2	22.0 ± 4.1	289.1 ± 76.2	88.6 ± 0.2	0.391 ± 0.001	−41.7 ± 0.2	98.3
CP3	22.2 ± 3.7	274.3 ± 73.9	93.3 ± 0.1	0.192 ± 0.000	−42.5 ± 0.2	98.9
CP4	22.3 ± 4.3	291.0 ± 75.5	95.0 ± 0.0	0.141 ± 0.001	−43.6 ± 0.3	99.4
CP5	21.7 ± 3.7	284.0 ± 73.8	95.3 ± 0.3	0.111 ± 0.000	−44.4 ± 0.0	99.5
CP6	21.9 ± 4.0	282.4 ± 55.1	95.8 ± 0.3	0.100 ± 0.000	−45.4 ± 0.4	99.6

^a^ Samples were coded according to cycles of HPH treatment. ^b^ The width and length of more than fifty particles in the supernatant were measured from TEM images using GMS 3 softeware. ^c^ A diluted CNCs suspension was centrifuged at 10,000 RPM for 30 min to separate CNCs from non-nanocellulose sediment. The sediment fraction was dried at 70 °C for 24 h in a vacuum oven dryer. The degree of isolation of the CNCs was calculated using the following equation: degree of isolation of CNCs (%) = (1− (weight of dried sediment)/weight of total solid)) × 100 [31]. ^d^ Turbidity was measured at 870 nm using a ultraviolet-visible spectrophotometry (UV-1650PC, Simadzu, Japan). A cuvette filled with DI water was used as blank. ^e^ The ζ-potential of CNCs suspensions was characterized with a ZetaSizer Nano ZS (Malvern Instruments, UK) utilizing electrophoretic light scattering at transmission of 25% [45]. ^f^ The centrifugal sedimentation experiments were carried out in a LUMisizer 610 (L.U.M. GMbH, Germany) at transmission of 25%. ^g^ The dimensions were measured using an optical microscope (BX51, OLYMPUS, China). The unit of size is a micrometer.

**Table 2:** Segal crystallinity index, crystallite size and degree of polymerization from diverse raw materials.

Materials	Crystallinity Index CI (%) ^a^	Crystallite Size τ (Å) ^b^	Degree of Polymerization (DP_V_) ^c^	Sulfur Content (mg/kg) ^d^
Microcrystallinecellulose (MCC)	82.0	6.7	348.0	8510
Softwood pulp (SWP)	85.2	4.3	1265.7	6490
Cotton pulp (CP)	91.6	6.3	1070.7	5740

^a^ The crystalline index was calculated using the diffraction intensities of the crystalline structure and that of the amorphous fraction according to the method of Segal [31]. ^b^ The dimension *D_hkl_* of the crystallites perpendicular to the *hkl* diffracting planes was estimated from the peak broadening in the XRD profiles after deconvolution, using Scherrer’s equation [30]. ^c^ Viscosity measurements of a cellulose sample dissolved in cupriethylenediamine (CED) were used to estimate the degree of polymerization. The relationship between the intrinsic viscosity and the degree of polymerization has been formulated as Eq. [49]. ^d^ The sulfur content of CNCs was analyzed using an ICP-AES (ICAP 7400, Thermo Scientific, MA, USA) to predict the degree of sulfation [50].

## Data Availability

Data are contained within the article.

## Supplementary Materials

The following are available online at https://www.mdpi.com/article/10.3390/ma14216463/s1, Figure S1: Representative AFM and TEM images of CNCs obtained by sulfuric acid hydrolysis of three different materials in accordance with high-pressure homogenizer treatment: (a) and (d) CNCs(MCC), (b) and (e) CNCs(SWP), (c) and (f) CNCs(CP), respectively, Figure S2: A scatter plot graph and correlation analysis results for the isolation degree of CNCs and suspension stability: CNCs(MCC); Square, CNCs(SWP); Circle, CNCs(CP); Triangle, Figure S3: Stability of CNCs suspensions accordance with multisample analytical centrifuge (LUMiSizer®) of the three samples at 23 °C. All stable samples are identified as such by a unvaryingness in the light transmission along the cuvette since the 1 or 2 passes of high-pressure homogenizer treatment.

## References

[1] Habibi Y., Lucia L.A., Rojas O.J. (2010). Cellulose nanocrystals: Chemistry, self-assembly, and applications. Chem. Rev..

[2] Peresin M.S., Habibi Y., Zoppe J.O., Pawlak J.J., Rojas O.J. (2010). Nanofiber Composites of Polyvinyl Alcohol and Cellulose Nanocrystals: Manufacture and Characterization. Biomacromolecules.

[3] Yang J., Han C.R., Zhang X.M., Xu F., Sun R.C. (2014). Cellulose Nanocrystals Mechanical Reinforcement in Composite Hydrogels with Multiple Cross-Links: Correlations between Dissipation Properties and Deformation Mechanisms. Macromolecules.

[4] Kanoth B.P., Claoudino M., Johansson M., Berglund L.A., Zhou Q. (2015). Bionanocomposites from Natural Rubber: Synergistic Effects of Functionalized Cellulose Nanocrystals as Both Reinforcing and Crosslinking Agents via Free-Radical Thiol-ene Chemistry. ACS Appl. Mater. Interfaces.

[5] Liu Y., Li Y., Yang G., Zheng X., Zhou S. (2015). Multi-Stimulus-Responsive Shape-Memory Polymer Nanocomposite Network Cross-Linked by Cellulose Nanocrystals. ACS Appl. Mater. Interfaces.

[6] Lee M., Heo M.H., Lee H., Kim Y.W., Shin J. (2017). Tunable softening and toughening of individualized cellulose nanofibers-polyurethane urea elastomer composites. Carbohydr. Polym..

[7] Domingues R.M.A., Gomes M.E., Reis R. (2014). The Potential of Cellulose Nanocrystals in Tissue Engineering Strategies. Biomacromolecules.

[8] Roman M., Dong S., Hirani A., Lee Y.W., Edgar K.J., Heinze T., Buchanan C.M. (2009). Cellulose Nanocrystals for Drug Delivery. Polysaccharide Materials: Performance by Design.

[9] Lin N., Dufresne A. (2014). Nanocellulose in biomedicine: Current status and future prospect. Eur. Polym. J..

[10] Li M.C., Wu Q., Song K., Qing Y., Wu Y. (2015). Cellulose Nanoparticles as Modifiers for Rheology and Fluid Loss in Bentonite Water-based Fluids. ACS Appl. Mater. Interfaces.

[11] Sun X., Wu Q., Lee S., Qing Y., Wu Y. (2016). Cellulose Nanofibers as a Modifier for Rheology, Curing and Mechanical Performance of Oil Well Cement. Sci. Rep..

[12] Cao J., Zhang X., Wu X., Wang S., Lu C. (2016). Cellulose nanocrystals mediated assembly of graphene in rubber composites for chemical sensing applications. Carbohydr. Polym..

[13] Galland S., Andersson R.L., Salajková M., Ström V., Olsson R.T., Berglund L.A. (2013). Cellulose nanofibers decorated with magnetic nanoparticles—synthesis, structure and use in magnetized high toughness membranes for a prototype loudspeaker. J. Matter. Chem. C..

[14] Olsson R.T., Azizi S.M.A.S., Salazar-Alvarez G., Belova L., Ström V., Berglund L.A., Ikkala O., Nogués J., Gedde U.W. (2010). Making flexible magnetic aerogels and stiff magnetic nanopaper using cellulose nanofibrils as templates. Nat. Nanotech. Lett..

[15] Wicklein B., Kocjan A., Salazar-Alvarez G., Carosio F., Camino G., Antonietti M., Bergström L. (2015). Thermally insulating and fire-retardant lightweight anisotropic foams based on nanocellulose and graphene oxide. Nat. Nanotech. Lett..

[16] Korhonen J.T., Hiekkataipale P., Malm J., Karppinen M., Ikkala O., Ras R.H.A. (2011). Inorganic Hollow Nanotube Aerogels by Atomic Layer Deposition onto Native Nanocellulose Templates. ACS Nano.

[17] Reid M.S., Villalobos M., Cranston E.D. (2017). Benchmarking Cellulose Nanocrystals: From the Laboratory to Industrial Production. Langmuir.

[18] Trache D., Huccin M.H., Haafiz M.K.M., Thakur V.K. (2017). Recent progress in cellulose nanocrystals: Sources and production. Nanoscale.

[19] Lee M., Heo M.H., Lee H., Lee H.H., Jeong H., Kim Y.W., Shin J. (2018). Facile and eco-friendly extraction of cellulose nanocrystals via electron beam irradiation followed by high-pressure homogenization. Green Chem..

[20] Saito T., Nishiyama Y., Putaux J.L., Vignon M., Isogai A. (2006). Homogeneous Suspensions of Individualized Microfibrils from TEMPO-Catalyzed Oxidation of Native Cellulose. Biomacromolecules.

[21] Chinga-Carrasco G. (2013). Optical method for the quantification of the fibrillation degree of bleached MFC materials. Micron.

[22] Shimizu M., Saito T., Nishiyama Y., Iwamoto S., Yano H., Isogai A., Endo T. (2016). Fast and Robust Nanocellulose Width Estimation Using Turbidimetry. Macromol. Rapid Commun..

[23] Yano H., Sugiyama J., Nakagaito A.N., Nogi M., Matsuura T., Hikita M., Handa K. (2005). Optically Transparent Composites Reinforced with Networks of Bacterial Nanofibers. Adv. Mater..

[24] Aulin C., Gällstedt M., Lindström T. (2010). Oxygen and oil barrier properties of microfibrillated cellulose films and coatings. Cellulose.

[25] Fukuzumi H., Saito T., Iwata T., Kumamoto Y., Isogai A. (2008). Transparent and high gas barrier films of cellulose nanofibers prepared by TEMPO-mediated oxidation. Biomacromolecules.

[26] Taheri H., Samyn P. (2016). Effect of homogenization (microfluidization) process parameters in mechanical production of micro and nanofibrillated cellulose on its rheological and morphological properties. Cellulose.

[27] Heo M.H., Lee H., Jeong J.S., Jeong H., Yuk J.S., Park S.H., Choi S.Q., Shin J. (2021). Hybrid Nanocelluloses Isolated through Electron-Beam Irradiation in the Wet State: Redispersibility in Water and Superstability for Pickering Emulsions. ACS Sustain. Chem. Eng..

[28] Zhao C., Zhang H., Zeng X., Li H., Sun D. (2016). Enhancing the inter-fiber bonding properties of cellulosic fibers by increasing different fiber. Cellulose.

[29] Banavath H.N., Bhardwaj N.K., Ray A.K. (2011). A comparative study of the effect of refining on charge of various pulps. Bioresour. Technol..

[30] Flauzino N.W.P., Putaux J.L., Mariano M., Ogawa Y., Otaguro H., Pasquini D., Dufresne A. (2016). Comprehensive Morphological and Structural Investigation of Cellulose i and II Nanocrystals Prepared by Sulphuric Acid Hydrolysis. RSC Adv..

[31] Besbes I., Alila S., Boufi S. (2011). Nanofibrillated Cellulose from TEMPO-Oxidized Eucalyptus Fibres: Effect of the Carboxyl Content. Carbohydr. Polym..

[32] Johnson R.K., Zink-Sharp A., Glasser W.G. (2011). Preparation and Characterization of Hydrophobic Derivatives of TEMPO-Oxidized Nanocelluloses. Cellulose.

[33] Tanaka R., Saito T., Isogai A. (2012). Cellulose Nanofibrils Prepared from Softwood Cellulose by TEMPO/NaClO/NaClO2 Systems in Water at PH 4.8 or 6.8. Int. J. Biol. Macromol..

[34] Pääkko M., Ankerfors M., Kosonen H., Nykänen A., Ahola S., Österberg M., Ruokolainen J., Laine J., Larsson P.T., Ikkala O. (2017). Enzymatic Hydrolysis Combined with Mechanical Shearing and High-Pressure Homogenization for Nanoscale Cellulose Fibrils and Strong Gels. Biomacromolecules.

[35] Rezayati C.P., Dehghani-Firouzabadi M., Afra E., Shakeri A. (2013). Rheological Characterization of High Concentrated MFC Gel from Kenaf Unbleached Pulp. Cellulose.

[36] Naganuma T., Kagawa Y. (1999). Effect of particle size on light transmittance of glass particle dispersed epoxy matrix optical composites. Acta. Mater..

[37] Naganuma T., Kagawa Y. (2002). Effect of particle size on the optically transparent nano meter-order glass particle-dispersed epoxy matrix composites. Compos. Sci. Technol..

[38] Rahmawan Y., Xu L., Yang S. (2013). Self-assembly of nanostructures towards transparent, superhydrophobic surfaces. J. Mater. Chem. A..

[39] Hamid S.B.A., Zain S.K., Das R., Centi G. (2016). Synergic Effect of Tungstophosphoric Acid and Sonication for Rapid Synthesis of Crystalline Nanocellulose. Carbohydr. Polym..

[40] Udomkichdecha W., Chiarakorn S. (2001). Factors to Predict the Fibrillation Tendency of Lyocell Fibers. J. Sci. Res. Chula. Univ..

[41] Elazzouzi-Hafraoui S., Nishiyama Y., Putaux J.L., Heux L., Dubreuil F., Rochas C. (2008). The Shape and Size Distribution of Crystalline Nanoparticles Prepared by Acid Hydrolysis of Native Cellulose. Biomacromolecules.

[42] Bondeson D., Mathew A., Oksman K. (2006). Optimization of the isolation of nanocrystals from microcrystalline cellulose by acid hydrolysis. Cellulose.

[43] Bai W., Holbery J., Li K.A. (2009). Technique for Production of Nanocrystalline Cellulose with a Narrow Size Distribution. Cellulose.

[44] Dobrovolskaia M.A., Patri A.K., Zheng J., Clogston J.D., Ayub N., Aggarwal P., Neun B.W., Hall J.B., McNeil S.E. (2009). Interaction of Colloidal Gold Nanoparticles with Human Blood: Effects on Particle Size and Analysis of Plasma Protein Binding Profiles. Biol. Med..

[45] Jiang J., Oberdörster G., Biswas P. (2009). Characterization of Size, Surface Charge, and Agglomeration State of Nanoparticle Dispersions for Toxicological Studies. J. Nanoparticle Res..

[46] Capadona J., Shanmuganathan K., Trittschuh S., Seidel S., Rowan S.J., Weder C. (2009). Polymer nanocomposites with nanowhiskers isolated from microcrystalline cellulose. Biomacromolecules.

[47] Araki J., Wada M., Kuga S., Okano T. (1999). Influence of surface charge on viscosity behavior of cellulose microcrystal suspension. J. Wood Sci..

[48] Araki J., Wada M., Kuga S., Okano T. (1998). Flow properties of microcrystalline cellulose sus- pension prepared by acid treatment of native cellulose. Colloids Surf. A..

[49] Evans R., Wallis A.F.A. (1989). Cellulose Molecular Weights Determined by Viscometry. J. Appl. Polym. Sci..

[50] Chen L., Wang Q., Hirth K., Baez C., Agarwal U.P., Zhu J.Y. (2015). Tailoring the Yield and Characteristics of Wood Cellulose Nanocrystals(CNC) Using Concentrated Acid Hydrolysis. Cellulose.

[51] Huang L., Wu Q., Wang Q., Wolcott M. (2019). Mechanical Activation and Characterization of Micronized Cellulose Particles from Pulp Fiber. Ind. Crops Prod..

[52] Ng H.M., Sin L.T., Tee T.T., Bee S.T., Hui D., Low C.Y., Rahmat A.R. (2015). Extraction of Cellulose Nanocrystals from Plant Sources for Application as Reinforcing Agent in Polymers. Compos. Part. B Eng..

