# Supplementary material for: Facile and Quantitative Method for Estimating the Isolation Degree of Cellulose Nanocrystals (CNCs) Suspensions
Source: Materials (Basel). 2021 Oct 28;14(21):6463. doi: 10.3390/ma14216463 (PMC8585153; doi:10.3390/ma14216463)
Supplement: Supplementary file 1 [file materials-14-06463-s001.zip › materials-1416933-supplementary.pdf]

materials
MDPI

Supplementary Materials

Facile and Quantitative Method for Estimating the Isolation Degree of Cellulose Nanocrystals (CNCs) Suspensions

Minwoo Lee [1,*,†], Minhaeng Heo [1,2,†], Hyunho Lee [1,2] and Jihoon Shin [1,2,*]

1 Center for Environment & Sustainable Resources, Korea Research Institute of Chemical Technology (KRICT), 141 Gajeong-ro, Yuseong-gu, Daejeon 34114, Korea; mhheo@krict.re.kr (M.H.H.); hyunho@krict.re.kr (H.L.)
2 Department of Advanced Materials & Chemical Engineering, University of Science & Technology (UST), 217 Gajeong-ro, Yuseong-gu, Daejeon 34113, Korea
* Correspondence: mwlee@hansol.com (M.L.); jshin@krict.re.kr (J.S.)
† These authors contributed equally to this work.

Contents of Supporting Information

Annexure S1	Experimental	Page S1–S3
Figure S1	AFM and TEM images	Page S3
Figure S2	A scatter plot graph	Page S3
Figure S3	Transmission profile analysis	Page S4

Experimental

Materials

Three different types of cellulosic materials, microcrystalline cellulose (MCC), bleached softwood kraft pulp (SWP) and cotton lint pulp (CP) were used as the starting material for the preparation of cellulose nanocrystals (CNCs). MCC (cellulose, microcrystalline, powder, Cat. No. 435236), sulfuric acid (H_2SO_4, 95%), sodium hydroxide (NaOH) and other reagents was purchased from Sigma-Aldrich(Burlington, STL, USA) and used without further purification. A commercial BSKP was used as a native wood cellulose sample, provided by Hansol Paper Co., Ltd.(Daejeon, Korea) Dissolving-grade CLP was kindly provided by Korea Minting and Security Printing Corporation (KOMSCO, Daejeon, Korea).

Preparation of CNCs via acid hydrolysis.

Sulfuric acid-hydrolyzed CNCs were prepared according to the typical and widely used method of Tang et al., with some minor modifications [1]. In brief, 60 g of the cellulose samples and 600 mL of distilled water mixture were put in an ice-bath and stirred gently for a while. 540 mL sulfuric acid was added dropwise to the cellulose/water mixture, and the hydrolysis reaction was performed at 40 °C for 2 hours under vigorous agitation. The acid was removed from the suspension by repeated centrifugation at 4 °C and 10,000 rpm for 10 min and terminated until a constant pH was observed. After the centrifugation and/or washing process, the sediment was collected and prepared suspension with a concentration of 1 wt%. The milky suspension obtained was processed through a high-pressure homogenizer (Nano DeBEE, BEE International, MA, USA) with a diamond process nozzle D5 (130 micron) under an operating pressure of 15,000 psi. The suspensions passed through the interaction chambers at a rate of 400 mL min^{-1} for 1 to 6 passes and the temperature during the homogenization process was maintained below 10 °C using a circulating-water cooling pump. After 6 passes, the obtained sample was stored at 4 °C for further use in turbidity, zeta potential, and TEM studies.

Separation by centrifugation

The performance of separation by size involved fractions from the CNCs suspension, isolated by homogenization, and was carried out using a centrifuge system. CNCs suspensions were centrifuged at 11,000 Rotational Centrifugal Force (RCF) for 30 min for 1 to 6 passes, and the resulting supernatant and sediment were respectively collected. The sediment was resuspended in water for turbidimetry analysis.

Calculation of nanosized fraction yield

The sediment fraction was dried at 70 °C for 24 h in a vacuum oven dryer. The CNCs fraction ratio of the CNC solution was calculated from [2] :

$$\text{Isolation degree of CNCs suspension (\%)} = \left(1 - \frac{Weight\ of\ dried\ sediment}{Total\ weight\ (supernatant\ fraction\ and\ sediment\ fraction)}\right) \quad (1)$$

Turbidity

CNC suspensions were put in a cuvette and the transmittance was measured at 870 nm using an ultraviolet-visible spectrophotometer (Simadzu, UV-1650PC, Kyoto, Japan). A cuvette filled with DI water was used as a blank.

Zeta Potential

To determine the surface charge of the CNCs suspensions, the suspensions were diluted to a concentration of 0.5–1.0 wt%. To determine the zeta potential of the CNCs suspensions, a Zetasizer (Malvern Panalytical, Zetasizer Nano ZS, Malvern, UK)was used. The instrument measures electrophoretic light scattering from a 35 mW solid state laser beam at a 660 nm wavelength. All reported values were an average of 5 measurements with confidence intervals of 95%.

Multisample analytical centrifuge (LUMISizer®).

The Multisample analytical centrifuge - LUMISizer® (L.U.M. GmbH, Berlin, Germany) was used in this study, which allowed measurement of the intensity of the transmitted light as a function of time and position over the entire sample length simultaneously. CNCs suspensions were transferred into a 2.0 mm optical path length polycarbonate (PC) disposable cell. The instrumental parameters used for the measurement were as follows: 1.8 mL of volume, 4000 rpm, 60 s of time intervals and 25 °C of condition temperature.

Atomic Force Microscopy (AFM)

The tapping mode AFM measurements were performed using a MultiMode 8® NanoScope system (Bruker, Billerica, MA, USA). Samples were prepared by spin coating a few drops of 0.001% (w/w) CNCs dispersion on a mica plate (1 × 1 cm) treated by 0.01% (w/w) poly-L-lysine solution (PLL) coating. After vacuum drying at 50 °C to remove residual water, the mica plate was observed.

Transmission Electron Microscopy (TEM)

The morphologies of different three different CNCs were observed with a JEOL JEM-3011(Jeol Ltd., Tokyo, Japan) transmission electron microscope (TEM) operated at an accelerating voltage of 200 kV. A drop of 0.01% (w/w) diluted CNCs dispersion was deposited on a thin-carbon-coated 200 mesh copper grid (CF200–Cu, EMS), which was negatively stained with 0.2% (w/w) uranyl acetate and then allowed to dry. The image was taken under diffraction contrast in the bright-field mode without prior contrast enhancement.

X-ray Diffraction Analysis (XRD)

Crystallinity measurements were performed on a Rigaku D/MAX-2200V equipment(Rigaku Corporation, Tokyo, Japan). The diffracted intensity of Cu Kα radiation (λ = 1.54 Å; 40 kV and 40 mA) was measured in a 2θ range between 5° and 40°. The samples were lyophilized at −50 ℃ for 4 days using a freeze dryer (TFD5503, Ilshinbiobase Co. Ltd., Korea) before measurement

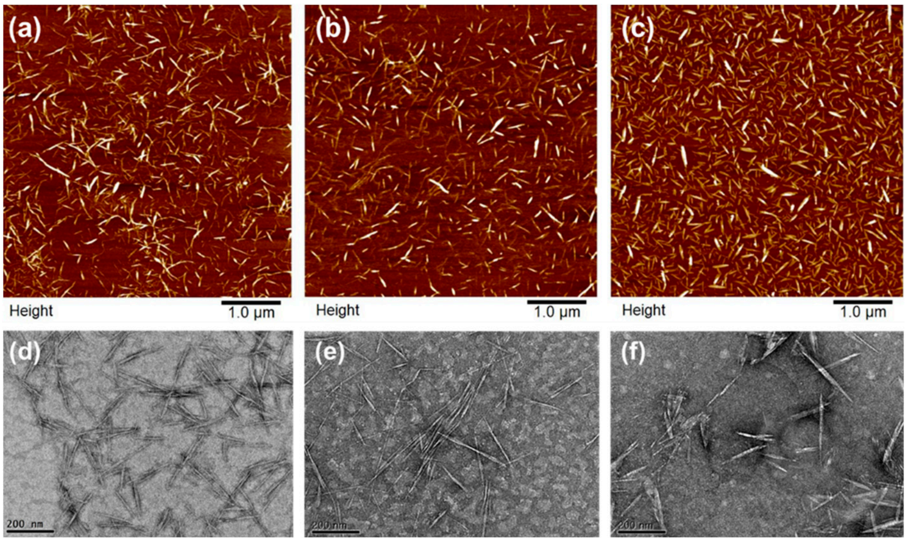

Figure S1. Representative AFM and TEM images of CNCs obtained by sulfuric acid hydrolysis of three different materials in accordance with high-pressure homogenizer treatment: (a) and (d) CNCs(MCC), (b) and (e) CNCs(SWP), (c) and (f) CNCs(CP), respectively.

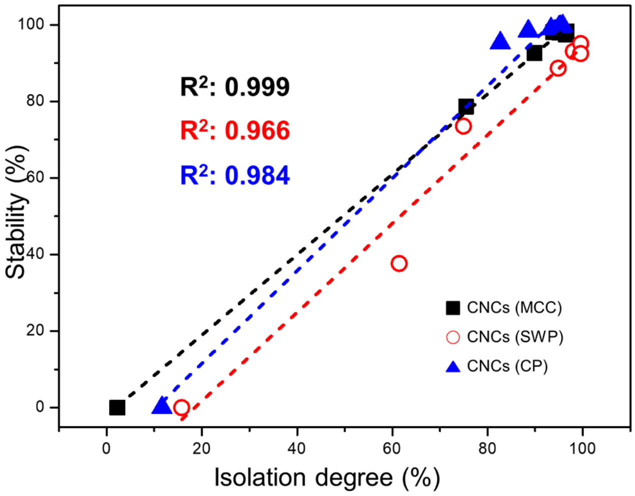

Figure S2. A scatter plot graph and correlation analysis results for the isolation degree of CNCs and suspension stability: : CNCs(MCC); Square, CNCs(SWP); Circle, CNCs(CP); Triangle.

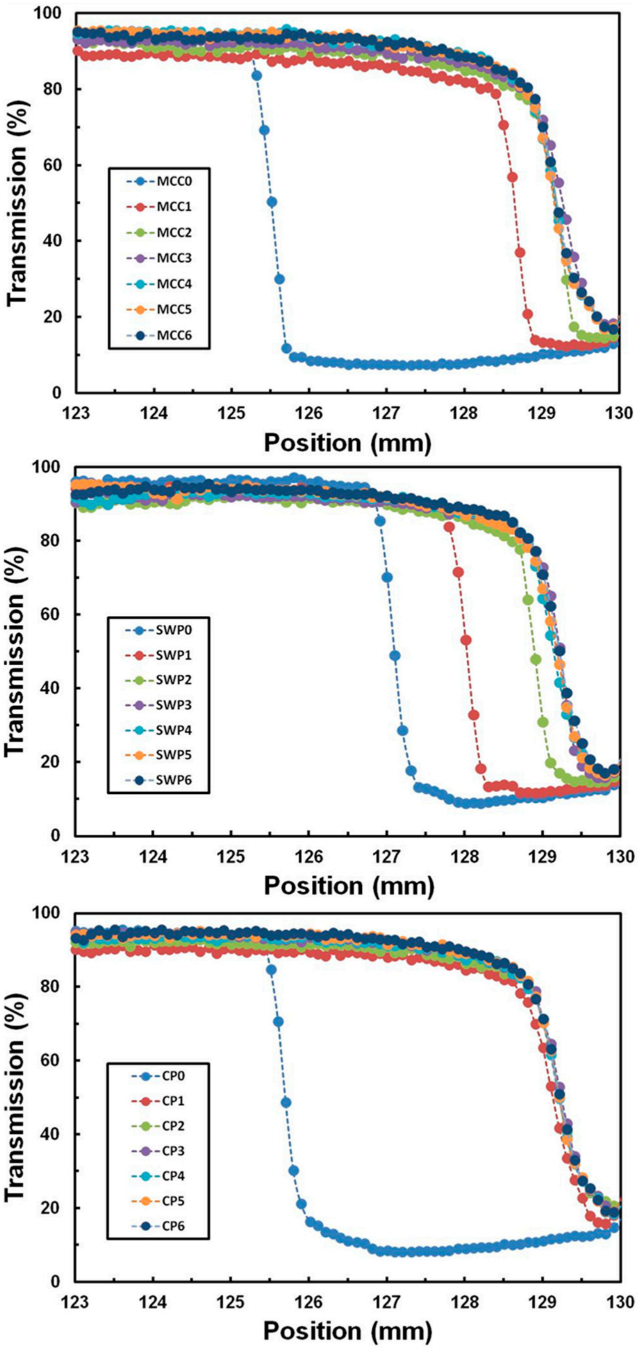

Figure S3. Stability of CNCs suspensions accordance with multisample analytical centrifuge (LUMiSizer®) of the three samples at 23°C. All stable samples are identified as such by a unvaryingness in the light transmission along the cuvette since the 1 or 2 passes of high-pressure homogenizer treatment.

References

1. Li-rong, T.; Biao, H.; Wen, O.; Xue-rong, C.; Yan-dan, C. Manufacture of cellulose nanocrystals by cation exchange resin-catalyzed hydrolysis of cellulose. *Bioresour. Technol.* **2011**, *102*, 10973–10977, doi:10.1016/j.biortech.2011.09.070.
2. Peresin, M.S.; Habibi, Y.; Zoppe, J.O.; Pawlak, J.J.; Rojas, O.J. Nanofiber Composites of Polyvinyl Alcohol and Cellulose Nanocrystals: Manufacture and Characterization. *Biomacromolecules* **2010**, *11*, 674–681, doi:10.1021/bm901254n.
